# Supplementary material for: Animal-Assisted Therapy Improves Communication and Mobility among Institutionalized People with Cognitive Impairment
Source: Int J Environ Res Public Health. 2020 Aug 14;17(16):5899. doi: 10.3390/ijerph17165899 (PMC7459682; doi:10.3390/ijerph17165899)
Supplement: Supplementary file 1 [file ijerph-17-05899-s001.pdf]

Supplementary Table S1. Objectives, exercises, and descriptions of the therapy dog participation in each session.

SESSION	OBJECTIVES	EXERCISES	THERAPY DOG PARTICIPATION
1	To work on the gait with short distances and changes of direction.	1. Walk up to the cone and back.<br>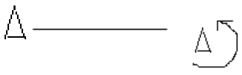<br>2. Walk around the cones forming an eight.<br>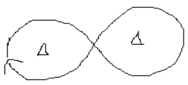<br>3. Communication: Pets.	1 and 2. The therapy dog walks with the participant during the movements.<br>3. The therapy dog is sitting in front of the participants and stays calm while they talk. Participants pet, brush, and feed the dog.
2	Perform throwing exercises to stimulate static balance.<br>Build a bond with the therapy dog.	1. In a standing position, throw objects (ball, rope, toys):<br>- Near / far / hit a hoop<br>- With the right and left hand<br>- With both hands<br>2. Communication: The weather	1. In all cases, the therapy dog returns the objects to the participant.<br>2. Participants pet, brush, and feed the dog.
3	Re-educate yourself from obstacles to encourage the aspect of lifting your feet off the ground.	Different circuits in the room:<br>1. Flat parallel rings<br>2. Flat zig-zag hoops<br>3. Hoop raised with cleats<br>4. Communication: Name different actions and relate them to day or night.	1, 2, and 3. The therapy dog accompanies the participants during the circuit. At the end of the circuit, there is a large ring where participants have to make the dog sit.<br>4. Participants pet, brush, and feed the dog.
4	Work height changes to stimulate static balance<br>Wander without dragging your feet.	1. Circuit with rings:<br>Go to each large hoop and bend down to catch the small hoop that is on the dog. On the way back, go to each large hoop, remove the small hoop from the dog and leave it on the ground.<br>2. Communication: Relate the days of the week with usual activities.	1. The therapy dog waits with the participants while performing the exercises.<br>.<br>2. Participants pet, brush, and feed the dog.
5	Imitate actions of daily life that imply changes of position and movements with	1. Imitation exercises:<br>Sit / get up<br>Turn on itself	1. The participants carry out the exercises with the therapy dog and must make the dog do the same (sit, turn, etc.).

	changes in speed and height.	From sitting / standing walk towards the cone<br><br>2. Communication: Try to remember what happens in each season of the year.	2. The therapy dog lies in front and the participant caresses the dog.
6	Work actions of daily life that involve height changes and turns.	1. Exercises to perform different actions: Go over a pike, bend down, stretch ...<br><br>2. Communication: Talk about traditional festivals. Relate the parties that are held in the season of the corresponding year.	1. Perform the exercises with the dog.<br><br>The therapy dog is located in the middle of the room and the participants are divided on each side of the dog and they throw the balls at each other over the dog.<br><br>2. Participants pet, brush, and feed the dog.
7	Work paths that involve constant changes of orientation to enhance rebalancing.	1. Circuit with cones:<br><br>Go zig-zag and come back in a straight line<br><br>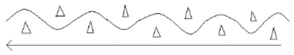<br><br>Go zig-zag, then through pikes, and come back in a straight line.<br><br>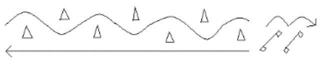<br><br>2. Communication: The parts of the body.	1. The therapy dog walks with the participant during the movements.<br><br>2. The therapy dog is sitting in front of the participants and stays calm while they talk. Participants pet, brush, and feed the dog.
8	Move around the room to work the march.	1. Different dog toys are hidden around the room at different heights. Participants must find and collect the toys.<br><br>2. Communication: Talk about toys, what they are, what material they are made of, colors, etc.	1. The therapy dog walks with the participants during the movements.<br><br>2. They will use the toys to play with the dog.
9-11	To promote aspects of the gait most frequently affected, such as dragging the feet, in circuits that involve lifting them	1. Different circuits will be made, for example:<br><br>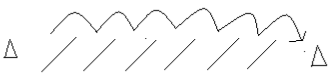<br><br>2. Communication: Talk about food, hobbies, news	1. The therapy dog walks with the participant during the movements.<br><br>2. The therapy dog is sitting in front of the participants and stays calm while they talk. Participants pet, brush, and feed the dog.

12	Make short trips exaggerating muscular actions involved in walking and increase the base of support to give greater stability.	1. Walk sideways to the end of the room.<br><br>Walk on your back to the end of the room.<br><br>2. Keep a standing position with legs apart.<br><br>3. Communication: Say goodbye.	1. The participant will walk to find the dog and put a collar on it.<br><br>2. While performing the exercise the therapy dog passes through the participants legs as a tunnel<br><br>3. Participants pet, brush, and feed the dog.

Supplementary Methods: Ilerkan internal health protocols.

Zoonosis prevention protocol

- Veterinary controls every three months
- Internal and external periodic parasite removal following the veterinarian indications
- General and specific annual tests (Leishmaniosis, filaria, Brucellosis, leptospirosis and ehrlichiosis)
- Stool tests every 45 days (between parasite removal periods)
- Annual vaccination: rabies, distemper, hepatitis, leptospirosis and parvovirus.
- Hygiene standards of therapy dogs: periodic bathing and grooming before each session.

Animal wellbeing protocol

- Stress prevention of the therapy dog previous, during and after the sessions.
- Positive training of the therapy dog
- Familiarisation of the therapy dog to technical aids, materials and healthcare context.
- Therapy dogs are integrated in our families therefore used to live with us and to follow the hygienic rules and cohabitation.
- Necessary physical exercise depending on the breed and age of each therapy dog.
- Mental and olfactory stimulation with detection sessions
- Maximum of two daily sessions (2 hours) respecting the resting periods between sessions, the weekend and one full month per year without taking part in any intervention.
- Therapy dog transport is done following the established road safety rules.
- Fulfilment of the regulations for pet possession: implanted chip, registered and identified with a plaque.
- Civil liability insurance during the execution of the tasks assigned to a therapy dog
- During sessions, the interaction between the patient and the therapy dog is controlled to ensure the interaction is positive and to prevent possible stressing situations

- The animal assisted intervention professionals who accompany the therapy dog have been trained in ethology and canine non-verbal communication to recognize and redirect any calm or stress signals that could take place
- Therapy dogs follow a balanced and high-quality diet appropriate for their needs
